# Relationship among number of close friends, subclinical geriatric depression, and subjective cognitive decline based on regional homogeneity of functional magnetic resonance imaging data

**DOI:** 10.3389/fnagi.2022.978611

**Published:** 2022-09-23

**Authors:** Zhao Zhang, Guangfei Li, Zeyu Song, Ying Han, Xiaoying Tang

**Affiliations:** ^1^Department of Biomedical Engineering, School of Life Sciences, Beijing Institute of Technology, Beijing, China; ^2^Department of Psychiatry, Yale University School of Medicine, New Haven, CT, United States; ^3^Department of Neurology, Xuanwu Hospital of Capital Medical University, Beijing, China

**Keywords:** number of close friends, subjective cognitive decline, regional homogeneity, mediation effect, subclinical geriatric depression

## Abstract

The relationship between geriatric depression and dementia has been widely debated, and the neurological mechanisms underlying subjective cognitive decline (SCD) associated with social relationships remain elusive. Subclinical geriatric depression (SGD) is common in patients with SCD, and close friends (CFs) have a great influence on a person’s social life. Studies have proven that communication or leisure activities with CFs can improve the cognitive performance of elderly. However, it remains unclear whether the engagement of specific brain regions mediates having CFs, SGD, and SCD. In this study, we aimed to assess the association between social relationships (that is, CFs), SGD, and SCD from the perspective of brain function. We examined the data of 66 patients with SCD and 63 normal controls (NC). Compared with NC, SGD was significantly inversely correlated with the number of CFs in the SCD group. We calculated regional homogeneity (ReHo) of functional magnetic resonance imaging (MRI) data of each subject. At a corrected threshold, the right occipital gyrus (SOG.R) and right fusiform gyrus (FFG.R) exhibited positive correlation with SGD in patients with SCD. Mediation analyses to query the inter-relationships between the neural markers and clinical variables exhibited a best fit of the model with CFs → FFG.R → SGD → SOG.R → SCD. These findings suggested a pathway whereby social relationships alter the function of specific brain regions, and SGD may be an early symptom of SCD. We observed that the FFG.R mediate social relationships and SGD, and the abnormality of the SOG.R may be a key factor in the SCD caused by depression. Moreover, a greater number of CFs may reduce the risk of developing SGD.

## Introduction

In the absence of verifiable neuropsychological abnormalities, subjective cognitive decline (SCD) refers to an individual’s perception of deterioration of memory and/or other cognitive skills compared with their former level of performance (Jessen et al., 2014). Evidence suggests that older people with SCD are more likely to have biomarker abnormalities associated with the pathology of Alzheimer’s disease (AD), as well as a higher risk of pathological cognitive decline (CD) and dementia in the future (Jessen et al., 2014; Jia et al., 2020). SCD may influence emotional and social functioning and general quality of life, in addition to being a precursor of non-normative CD (Jenkins et al., 2015). Effective interventions to postpone or prevent pathological CD may be best addressed in the early symptomatic illness stages while cognitive performance is still reasonably intact. Therefore, it is critical to identify the neuropathogenesis of SCD for the early identification of people at risk of developing AD and dementia.

One of the most well-studied risk factors for AD is depression. Epidemiological investigations have reported a putative link between depression and AD. However, related findings are inconsistent, perhaps because of the difficulty in distinguishing between early AD and late-onset depression. Some case-control studies have reported a link between a history of depression and the risk of AD (Tsolaki et al., 1997; Yang et al., 2020). However, some of these studies may have certain biases that make them difficult to analyze. Patients with AD, who frequently strive to remember their prior experiences more meticulously than control participants, may be skewed by a stronger recall of their history of depression in case-control studies that are essentially retrospective. Furthermore, some case-control studies reported negative results (Broe et al., 1990; Zalsman et al., 2000). Therefore, no definite link exists between depression and the chance of developing AD later in life. Other study-related variables may have influenced the results of these investigations. Some studies on depression and AD investigated the link between the frequency of depression symptoms and an AD diagnosis, whereas other studies investigated the link between symptoms and cognitive function tests including the Mini Mental State Examination rather than an AD diagnosis.

Subclinical depressive symptoms have been proven to increase the likelihood of cognitive impairment in elderly people without objectively measured cognitive decline (Balash et al., 2009). Since SCD may not be related to current cognitive performance, depressive symptomatology should be taken into consideration when patients appear at the clinic with subjective cognitive problems (Zlatar et al., 2014). Increased subclinical depressive symptoms are commonly linked to early cognitive impairment (Sun et al., 2008) or later dementia (Kaup et al., 2016) and may be a prodromal indicator of AD (Bierman et al., 2007). However, arguments can be made that depressive symptoms exacerbate SCD and, vice versa, that SCD contributes to the development of affective symptomatology (Hill et al., 2016). Evidence from long-term studies shows that people with SCD have a higher risk of depression, although further research is required to fully understand the temporal correlations between these factors. A person’s view of their cognitive state may be influenced by depression symptoms (Cargin et al., 2008). Alternatively, for older people who are frequently concerned about losing their autonomy, seeing a memory decline may have a negative impact on their psychological and emotional wellbeing (Quine and Morrell, 2007). Overall, the data currently available consistently supports links between SCD and subclinical depressive symptoms across studies, although little is known about the timing of these connections.

Currently, depression is thought to be a collection of syndromes generated mostly by biological and social variables, as well as interpersonal stress and other broad causes, and exhibited primarily as psychological abnormalities and associated disorders. Recent studies have connected emotions of belonging, social support, conflict, and loneliness to depression, despite the fact that most of the studies have focused on biological reasons. Hagerty and Williams (1999) reported that the feeling of belonging is the experience and capacity of a human system to interact with social and environmental factors, as well as interpersonal relationships. People who have a strong feeling of belonging believe that they are a vital part of their society and surroundings; therefore, they believe that they can actively engage and communicate. One of the most fundamental human motives is a sense of belonging, which is directly linked with psychological and social functions. A strong feeling of belonging may encourage the human body to perform at its best. The structural elements of social groups, as well as the capacity to seek help and enjoyment, are aspects of social support. The onset, course, and outcome of depression are influenced by social support.

Early studies have reported that the social environment of an individual, especially social interactions, might influence behavior and mental health (Mama, 2020). A longitudinal cohort study examining the link between loneliness and the risk of AD reported that those who lived alone exhibited a higher risk of developing AD than those who did not (Wilson et al., 2007). Unexpectedly, brain autopsy revealed that loneliness score was unrelated to immunoreactive plaques or cerebral infarction, which was inconsistent with the reported direct contribution of social isolation to the risk of developing AD and implied that the underlying mechanism is not related to the pathology of AD (Wilson et al., 2007). In contrast, a subsequent investigation using the Pittsburgh Compound B-PET criteria to quantify fibrillary amyloid burden in cognitively normal elderly people reported a link between loneliness and higher cortical amyloid, indicating that loneliness is a risk factor for preclinical AD (Donovan et al., 2016). Therefore, the neurobiological processes underlying memory loss linked to social isolation remain unknown.

Although social isolation is a complex and multifactorial concept, the number of close friends (CFs) is a very important influencing factor. Close friendships may assist in motivation to do exercise in later life. A person’s cognitive ability may be improved, allowing him to maintain his cognitive performance level in later life (Ihle et al., 2018). In this study, we hypothesized a significant association also existed between subclinical geriatric depression (SGD) and SCD because they are the preclinical stages of depression and AD, and we aimed to explore the neural mechanisms among the social relationships of the individuals affected by number of CFs, SGD, and SCD and to provide theoretical support for neuroimaging for better prevention and treatment of AD. We hypothesized that the number of CFs, SGD, and SCD are affected by a series of neural activities in the brain.

## Materials and methods

### Participants

In this study, we enrolled 66 patients with SCD and 63 normal controls (NC) from the Department of Neurology of Xuanwu Hospital of Capital Medical University. This study was approved by the Xuanwu Hospital Review Committee and the Medical Research Ethics Committee. Informed consent was obtained from all subjects.

The following were the SCD inclusion requirements: (1) self-perceived ongoing memory loss that is unrelated to an acute incident, such as a relative’s death or job loss; (2) self-concerns or worries about memory loss; and (3) performance on standardized neuropsychological tests within the normal range as adjusted by age, gender, and education. The requirements for NC were as follows: (1) No self-reported memory decline; (2) normal performance on common neuropsychological tests; and (3) memory complaints but no concerns.

SCD and NC exclusion criteria included: (1) MCI, dementia, or any other neurological diseases (such as Parkinson’s disease, epilepsy, or encephalitis) that might result in memory issues; (2) a history of stroke; (3) significant psychiatric conditions that impair cognition, such as depression with Hamilton Depression Scale (HAMD) > 24; (4) systemic conditions (such as syphilis, thyroid disorders, severe anemia, or HIV) that could impair cognition; (5) alcohol or drug abuse or dependence; (6) severe vision or hearing impairment; (7) failure to pass neuropsychological tests; and (8) MRI contraindications.

### Testing procedures

Data collection parameters were similar to those described previously (Supplementary Material) (Dong et al., 2020). All participants completed a conventional clinical evaluation that included a review of their health and family history, current medicines, physical examination, and standard blood tests, as well as evaluation using a battery of neuropsychological tests (Supplementary Material).

CFs (excluding spouse and child) is defined by the Model of interdependence (Levinger and Snoek, 1972): First, two people have a long time of frequent interaction. Second, there are various types of activities or events in this relationship, sharing many common activities and interests. Third, they have great influence on each other. Number of CFs was collected with a questionnaire, and it was based on individual reports, not including family informants.

### Data preprocessing

The quality of magnetic resonance imaging (MRI) scans was examined by experienced neuroradiologists, and images with artifacts were excluded. All MRI data were preprocessed using the standard method via the MATLAB-based DPARSFA (Yan et al., 2016) toolkit. This preprocessing mainly involved the conversion of the original data format (DICOM) to a usable format and included the removal of data for the first 10 time points, slice time correction, head motion correction, coregistration of anatomical and functional scans, spatial normalization into MNI space, low-frequency filtering, spatial smoothing with a 4-mm Gaussian kernel, and linear drift removal.

### Regional homogeneity

Regional Homogeneity (Zang et al., 2004) (ReHo) refers to the similarity between the time series (BOLD signal) describing a given voxel and BOLD time series of the nearest voxel. The voxels in this region are more uniform in time, which is a proven and reliable feature of resting-state functional MRI (fMRI). Consistency is described using Kendall’s consistency coefficient (KCC), which can measure the consistency trend of multiple sequences. If the time series are synchronized, KCC will approach 1 (ReHo approaches 1) and vice versa (ReHo approaches 0). DPARSFA (Yan et al., 2016) was used to calculate the ReHo brain map of each participant, and the algorithm was shown in Supplementary Material.

Voxel-wised whole brain multiple regression was performed against number of CFs, Geriatric depression scale (GDS), and SCD questionnaire of 9-item (SCD-9) using Statistical Parametric Mapping 12 tool (SPM12) (Ashburner and Friston, 2005) with clinical variables and ReHo images, in which age, gender, and years of education were used as covariates. The results were evaluated by combining voxel *p* < 0.001, uncorrected, with cluster *p* < 0.05 FWE-corrected for multiple comparisons. All voxel activations were reported in MNI coordinates. REST was used to extract the mean ReHo value of the activated regions. We performed further analysis using all the active brain regions, and GDS-related brain regions were identified after correlation analysis and mediation analysis. IBM SPSS Statistics version 24.0 (SPSS Inc., Chicago, IL, United States) was used for statistical analysis. Student’s *t*-test was used for inter-group analysis. Pearson correlation analysis was used to measure the correlation between two parameters.

### Mediation analyses

Mediation assessments (MacKinnon et al., 2007; Wager et al., 2008) were implemented for exploring the influences of independent variable X on dependent variable Y, obtaining the actual or theoretical association between X and Y, and then trying to explore the internal mechanism or principle of the relationship between X and Y. That is, we analyzed the influences of independent variable X on dependent variable Y. If variable X influences variable Y through affecting variable M, variable M is an intermediate variable. We used mediation assessments to study the correlations between neural markers and scale parameters across the subjects.

The mediation effect was analyzed using the PROCESS v4.1 toolkit (Hayes, 2013). Parameter checking was performed using the bias-corrected percentile bootstrap CI method. The principle of the algorithm is that N samples are first sampled randomly and repeatedly for N times; further, estimated value E of the mediation effect is calculated based on the N samples. Finally, the above steps are repeated M times (5,000 times in this paper), and the estimated values of these M mediation effects are taken as the point estimate value of the mediation effect. The M estimated values were sorted to obtain sequence C, and the 95% confidence interval of the mediation effect was estimated using the 2.5 percentile and 97.5 percentile of sequence C.

## Results

### Demographic characteristics

Demographic details are summarized in Table 1.

**TABLE 1:** Group characteristics and subject demographics.

	SCD	NC	t or χ^2^	p
	*N* = 66	*N* = 63		
Age (year)	65.8 ± 5.17	64.3 ± 5.56	1.6399	0.7734
Gender (female/male)	41/25	32/31	χ^2^ = 1.684	0.1944
Years of education	11.7 ± 2.93	11.9 ± 3.33	–0.2656	0.7244
BMI	24.4 ± 2.61	25.2 ± 2.80	–1.7593	0.6682
MES	88.53 ± 7.17	89.68 ± 7.26	–0.9070	0.9882
GDS	3.44 ± 3.11	1.76 ± 1.59	–3.8290	**<0.0001**
CFs	5.32 ± 2.37	5.46 ± 2.34	0.3423	0.7327
SCD-9	5.30 ± 1.82	3.10 ± 2.19	–6.2313	**<0.0001**
MMSE	28.7 ± 1.22	28.7 ± 1.29	0.0556	0.3860

MES, Memory and executive function screening instrument; GDS, Geriatric depression scale; CFs, Close friends; SCD-9, Subjective cognitive decline questionnaire of 9-item; MMSE, Mini–mental state examination. Bold values mean a significant difference exists between SCD and NC.

**FIGURE 1:**
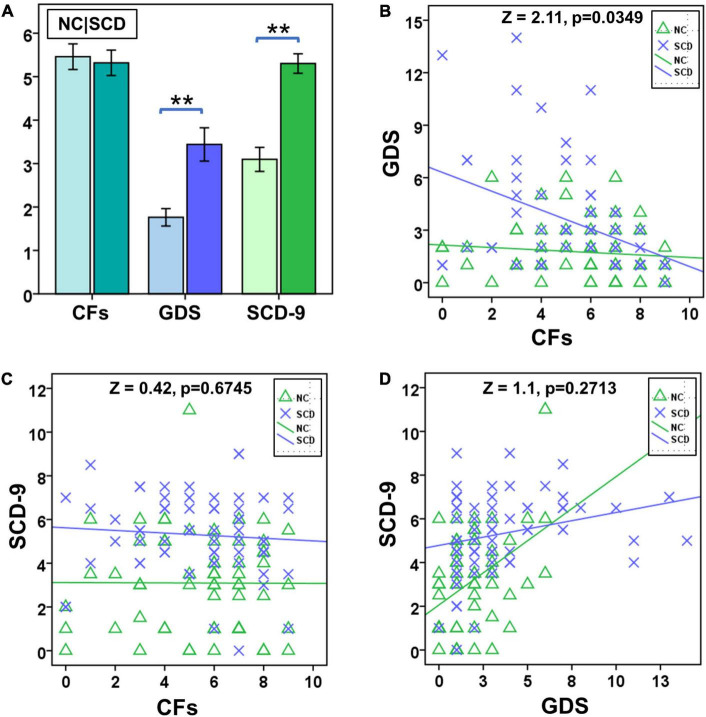
Statistical analysis of number of close friends (CFs), geriatric depression scale (GDS), and subjective cognitive decline questionnaire of 9-item (SCD-9) between NC and SCD. **(A)** Between NC (the left one) and SCD (the right one), number of CFs exhibited no significant difference, whereas GDS and SCD-9 did. **(B)** The factor CF was significantly correlated with GDS in the SCD group. **(C)** The NC and SCD groups exhibited no significant correlation between number of CFs and SCD-9. **(D)** The NC and SCD groups exhibited significant correlation between GDS and SCD-9. **p < 0.01.

No significant difference was observed between NC and SCD in terms of number of CFs (*t* = 0.3423, *p* = 0.7327), whereas significant differences were observed between GDS (*t* = -3.8290, *p* < 0.0001) and SCD-9 (*t* = -6.2313, *p* < 0.0001; Figure 1A). In the NC group, no significant correlation existed between Number of CFs and GDS (*r* = -0.1042, *p* = 0.4164, Figure 1B) and between Number of CFs and SCD-9 (*r* = -0.0040, *p* = 0.9754, Figure 1C). GDS was significantly correlated with SCD-9 (*r* = 0.4264, *p* < 0.001, Figure 1D). In the SCD group, Number of CFs significantly correlated with GDS (*r* = -0.4111, *p* < 0.001, Figure 1B). Number of CFs exhibited no significant correlation with SCD-9 (*r* = -0.0778, *p* = 0.5345, Figure 1C), whereas GDS was significantly correlated with SCD-9 (*r* = 0.2574, *p* = 0.0369, Figure 1D).

Significant difference existed between NC and SCD in the slope test of Number of CFs and GDS (*Z* = 2.11, *p* = 0.0349, Figure 1B), whereas no significant difference was observed between NC and SCD in the slope test of Number of CFs and SCD-9 (*Z* = 0.42, *p* = 0.6745, Figure 1C), as well as GDS and SCD-9 (*Z* = 1.10, *p* = 0.2713, Figure 1D).

### Brain activations in regional homogeneity

**FIGURE 2:**
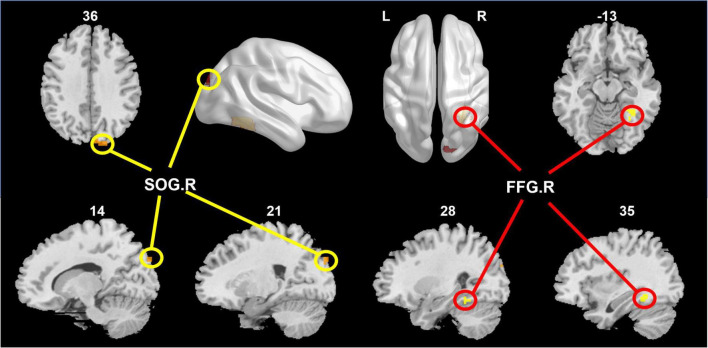
The right superior occipital gyrus (SOG.R) and right fusiform gyrus (FFG.R) were positively correlated with GDS in the SCD group.

After correcting family-wise error of multiple comparisons, GDS in the SCD group exhibited positive correlation in the right superior occipital gyrus (SOG.R; *x* = 18, *y* = -90, *z* = 36; peak voxel *T*-value = 5.27, volume = 27 mm^3^) and right fusiform gyrus (FFG.R; *x* = 33, *y* = -48, *z* = -12; peak voxel *T*-value = 5.47, volume = 25 mm^3^). Complete experimental results are given in Figure 2.

**FIGURE 3:**
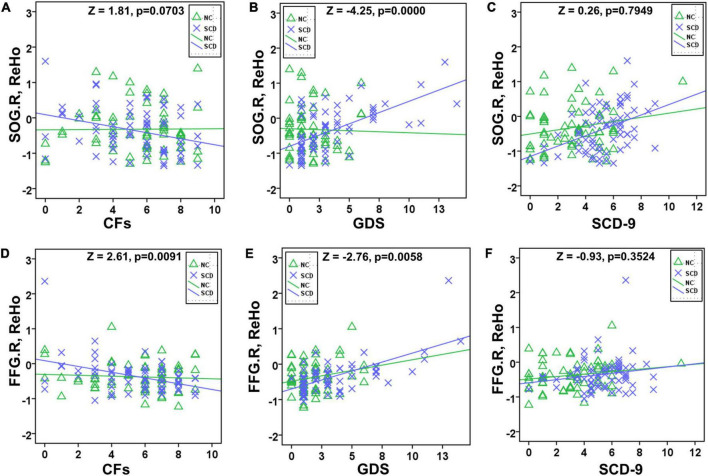
Slope test between NC and SCD in SOG.R/FFG.R with CFs/GDS/SCD-9. Significant difference existed between NC and SCD in GDS–SOG.R **(B)**, CFs–FFG.R **(D)**, and GDS–FFG.R **(E)**. No significant difference existed between NC and SCD in CFs–SOG.R **(A)**, SCD-9–SOG.R **(C)**, and SCD-9–FFG.R **(F)**.

The correlation of activated brain regions with number of CFs, GDS, and SCD-9 in the NC and SCD groups was compared (Figure 2). In the NC group, no significant correlation was observed in CFs–SOG.R (*r* = 0.0105, *p* = 0.9349, Figure 3A), GDS–SOG.R (*r* = –0.0277, *p* = 0.8296, Figure 3B), SCD–9-SOG.R (*r* = 0.1985, *p* = 0.1189, Figure 3C), CFs–FFG.R (*r* = -0.0695, *p* = 0.5881, Figure 3D), GDS– FFG.R (*r* = 0.2284, *p* = 0.0717, Figure 3E), and SCD-9–FFG.R (*r* = 0.1836, *p* = 0.1497, Figure 3F). In the SCD group, no significant correlation was exhibited between SCD-9 and FFG.R only (*r* = 0.1701, *p* = 0.1720, Figure 3F), whereas others exhibited significant correlation: CFs–SOG.R (*r* = -0.3069, *p* = 0.0122, Figure 3A), GDS–SOG.R (*r* = 0.6076, *p* < 0.001, Figure 3B), SCD-9–SOG.R (*r* = 0.4130, *p* < 0.001, Figure 3C), CFs–FFG.R (*r* = -0.4029, *p* < 0.001, Figure 3D), and GDS–FFG.R (*r* = 0.6474, *p* < 0.001, Figure 3E).

In the slope test, a significant difference existed between NC and SCD in GDS–SOG.R (*Z* = 2.11, *p* = 0.0349, Figure 3B), CFs–FFG.R (*Z* = 2.11, *p* = 0.0349, Figure 3D), and GDS–FFG.R (*Z* = 2.11, *p* = 0.0349, Figure 3E), whereas no significant difference existed between NC and SCD in CFs–SOG.R (*Z* = 0.42, *p* = 0.6745, Figure 3A), SCD-9–SOG.R (*Z* = 1.10, *p* = 0.2713, Figure 3C), and SCD-9–FFG.R (*Z* = 0.42, *p* = 0.6745, Figure 3F).

### Mediation model of brain regional response, close friends, subclinical geriatric depression, and subjective cognitive decline

**FIGURE 4:**
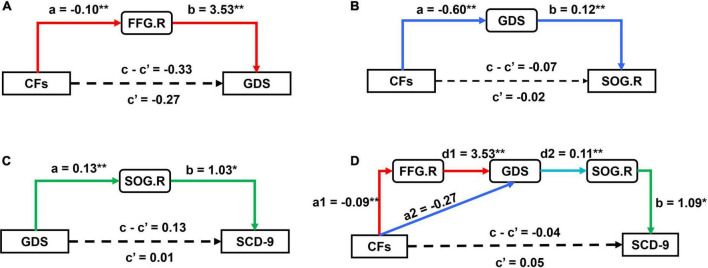
Results of mediation analysis between CFs, GDS, SCD-9, FFG.R, and SOG.R. **(A)** The model (CFs → FFG.R → GDS) exhibited a substantial (and complete) mediation; **(B)** the model (CFs → GDS → SOG.R) exhibited a substantial (and complete) mediation; **(C)** the model (GDS → SOG.R → SCD-9) exhibited a substantial (and complete) mediation; **(D)** the model (CFs → FFG.R → GDS → SOG.R → SCD-9) and (CFs→ GDS → SOG.R → SCD-9) both exhibited a substantial (and complete) mediation; other models are reported in Supplementary Figures 1–3. (*p < 0.05, **p < 0.01).

According to the above statistical analysis, CFs, FFG.R, and GDS; CFs, SOG.R, and GDS; and GDS, SOG.R, and SCD-9 were pairwise correlated in SCD group. Therefore, we conducted mediation analysis for these three groups of parameters with age, gender, and years of education as covariates. Each group of parameters had 6 mediation models (Supplementary Figures 1–3). In these three sets of models, the substantial (and complete) mediated relationship was preserved (Figures 4A–C). Further, we performed the chain mediation analysis with these three sets of mediation relationships (Figure 4D). The indirect effect and 95% confidence interval of the mediation effect of the four models are given in Table 2. If the confidence interval includes 0, there is no mediation relationship; if the confidence interval does not include 0, there is a substantial (and complete) mediation relationship.

**TABLE 2:** The indirect effect value and 95% confidence interval of the mediation effect of the four models.

Mediation relation	Indirect effect (X on Y)	BootLLCI (95%)	BootULCI (95%)
CFs→FFG.R→GDS	–0.33	–0.6096	–0.0716
CFs→GDS→SOG.R	–0.07	–0.1344	–0.0287
GDS→SOR.G→SCD-9	0.13	0.0400	0.2490
CFs→FFG.R→GDS→SOG.R→SCD-9	–0.04	–0.0889	–0.0045

## Discussion

**FIGURE 5:**
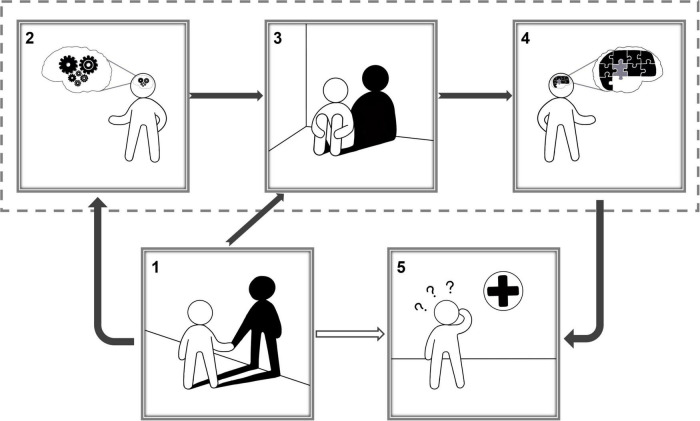
Schematic diagram of the conclusions of this paper, corresponding to the chain mediation model (1). A lonely person with only his/her own shadow for company; (2) the number of close friends affected a person’s brain activity in specific regions; (3) a depressed person; According to the chain mediation model, the number of close friends affects depression by influencing brain activity, and the number of close friends also directly affects depression; (4). Depression affects a person’s brain activity in specific regions; (5) a person suffering from subjective cognitive decline goes to the hospital; According to the chain mediation model, changes in activity in specific brain regions have an impact on cognitive function.

In this study, no significant differences were observed in age, gender, years of education, body–mass index, and number of CFs between SCD and NC, but they were observed between GDS andSCD-9. Combined with fMRI data based on ReHo, brain functional activity research is of great significance for studying normal aging and the physiological and pathological processes of SCD. The number of CFs is an important factor in determining loneliness in the elderly. In this study, an inverse relationship existed between the number of CFs and SGD in SCD group, that is, the more the number of CFs, the lower the risk of depression in the elderly. At the same time, a positive relationship between SGD and subjective cognitive function change was observed, that is, the higher the degree of SGD, the higher subjective decline in cognitive function. These findings highlight the consistency of the association between symptoms of depression and SCD (Zlatar et al., 2014, 2017). Numerous previous studies have confirmed the relationships between loneliness and depression separately (Cacioppo et al., 2010; Wan Mohd Yunus et al., 2013; Gan et al., 2015). These studies performed in the United States, Malaysia, and China reported that loneliness can independently positively predict depression among the elderly. The lonelier the elderly person, the more obvious the depression degree. In this study, we performed statistical analysis and explored the relationship of brain with the number of CFs, SGD, and cognitive function from neuroimaging perspectives. It is observed that the number of CFs in patients with SCD affects the function of the FFG.R region of the brain. The function change of FFG.R aggravates the occurrence of SGD, and SGD will lead to the function change of SOG.R and finally affect the severity of SCD. However, there is no such relationship in the NC group. Figure 5 shows a schematic diagram of the conclusions of this paper, describing the pathways of number of CFs, FFG.R, SGD, SOG.R, and SCD. The fundamental observations are discussed below.

### Emotion recognition of the fusiform gyrus: A mediator between the number of close friends and subclinical geriatric depression

Good relationships necessitate effective communication, which necessitates the effective exchange of information between two people. Mehrabian (1972) claimed that 55% of the information in speech comes from visual signals (facial expressions and gestures). Facial expression is a universal language for effective communication, and there is a high degree of consistency in the interpretation of facial expression in different countries or cultures. Human emotions of all kinds can be very sensitive to reflection through facial expressions. Facial expression change is very rapid, agile, and meticulous and can truly and accurately reflect the feelings and the transmission of information. This fully demonstrates the importance of emotion recognition in social relationships.

The FFG is a brain region that helps in recognizing faces, bodies, and bodily parts (McCarthy et al., 1997; Weiner and Grill-Spector, 2012; Zhu et al., 2019). In particular, face recognition is known to depend upon the neural activity of FFG (Henson et al., 2000; Rypma et al., 2015). Individual identity is vital for sustaining functioning social relationships (Yardley et al., 2008; Garrido-Vásquez Schmidt et al., 2011), a characteristic linked to improved recovery from depression and psychopathological resilience (George et al., 1989). A subclinical feature, known as alexithymia, is a transdiagnostic risk factor for mental illnesses. It affects approximately 20% of the depression patient population. Alexithymia is characterized by difficulties in defining and recognizing emotions, as well as an outwardly focused emotion-processing style, such as avoiding emotionally intense talks. Furthermore, facial recognition is a key aspect in emotion perception, which is decreased in patients with alexithymia (Lane et al., 2000). Patients with depression have a distinct link between alexithymia and brain anatomy compared with healthy controls. The FFG volume was linked to alexithymia in both patients and controls (Förster et al., 2019). Higher alexithymia scores on the FFG were linked to higher volume of gray matter in healthy controls and lower volume of gray matter in patients with depression. To the best of our knowledge, there have been no studies related to subclinical depression, FFG, and alexithymia in the subclinical depression stage, but Kuhn et al. (2019) emphasized the significance of psychoaffective elements at this point, including both subclinical anxiety and depression. Psychoaffective variables may not just be complicating factors in SCD; rather, they may be the first signs of the Alzheimer’s clinical syndrome or possibly the manifestation of a pathogenic process linked to psychological discomfort and eventual cognitive deterioration in SCD.

### Subclinical geriatric depression increases the risk of subjective cognitive decline, which is mediated by the superior occipital gyrus

An association between subjective cognitive complaints and symptoms of depression has been proven to exist (Hohman et al., 2011; Zlatar et al., 2014). In those with minimal to moderate levels of self-reported depressive symptoms as well as in people with considerable cognitive impairment (to the level of MCI), the link between SCD and depression symptoms was clear. It was also seen in those with both low and high SCD levels (Zlatar et al., 2017). These results suggest that when patients appear with SCD in a clinical environment, doctors should evaluate and take into consideration these symptoms. Depression is linked to an increased risk of AD (Gracia Garcia et al., 2013; Kuo et al., 2020), with recurrent depression episodes being especially harmful (Dotson et al., 2010). In addition, depression in middle and late life is considered to not only increase the risk of AD but also accelerate the onset and development of AD symptoms and degeneration, particularly in those with MCI (Barnes et al., 2012; Lee et al., 2012; Spalletta et al., 2012; Brendel et al., 2015; Gallagher et al., 2018). The prodromal aspect of AD may have a role in the onset of late-life depression. For example, executive dysfunction is frequent in both AD and late-life depression (Firbank et al., 2015), and it leads to the development of clinically substantial cognitive impairment. However, not everyone with the “depression-executive dysfunction syndrome” develops AD (Alexopoulos et al., 2002; Morimoto et al., 2015). Visual recognition is linked with the occipital lobe, which is also thought to play a role in episodic memory consolidation (Kukolja et al., 2016). The anatomical or functional alterations of the occipital lobe have been linked to depression (Liu D.-Y. et al., 2021). In the digit span backward task, Hilbert et al. (2019) employed repeated transcranial magnetic stimulation (rTMS) to investigate the use of visual processing techniques. Visualizers demonstrated a considerably higher mean performance loss after rTMS to the right occipital cortex than verbalizers, indicating that the virtual lesion to the right occipital brain is firmly linked with visual working memory processing (Pasternak and Greenlee, 2005; Sun et al., 2005; Suchan et al., 2006). Wu et al. (2013) found that the functional connectivity between SOG.R and posterior cingulated cortex increased in AD patients through resting-state fMRI data. Frankenberg et al. (2021) used factor analysis to investigate regional glucose uptake in specific brain regions for underlying subdimensions. They found that patients with AD showed lower values than controls on the factor occipital cortex. Studies on AD and depression have attracted extensive attention. Although the causes of AD are many, the SOG abnormality is well documented to lead to memory impairment, which is consistent with our hypothesis. Our statistical analysis and mediation model provided strong evidence that SGD may increase the risk of SCD, and the SOG.R plays a crucial role in the process.

### Subjective cognitive decline individuals with subclinical geriatric depression symptoms are more sensitive to social relationships

In the SCD group, the higher the number of CFs, the lower the severity of SGD (Figure 1B). However, this phenomenon was not seen in the NC group. This suggests that the number of CFs seems to be a particular factor in SCD with SGD symptoms. The number of CFs is a comprehensive measure of the quality and quantity of a person’s social relationships and a sign of a person’s active participation in social activities. There is no doubt that active social participation and interpersonal interaction can improve the quality of life; however, currently available studies have not reported the generality of this promoting effect, that is, social participation only occurs after a long period of time, such as years or decades. Afterward, it is not clear if the cognitive benefit accrue or whether its positive effect on cognition is immediate. SCD individuals with SGD symptoms are more sensitive to social relationships. The reason could be that people with SCD may already have changes in brain function (Li et al., 2021; Liu Y. et al., 2021). More social and emotional support is linked to more social activities and better brain functioning, while a lack of social and emotional support is linked to fewer positive relationships and less engagement in social activities, which results in less brain stimulation and a greater degree of depression (Wang et al., 2002; Schwarzbach et al., 2014; Weng et al., 2020). Long-term longitudinal studies investigating social engagement and cognitive function have reported that an active lifestyle in midlife can help in maintaining good cognitive function and reduce the risk of dementia in old age (Chang et al., 2010), which indicates that social participation would have a cumulative effect on cognitive function and there may be a measure-effect relationship between them.

### Limitations of the study and conclusion

This study has some limitations. First, the gold standard to investigate a causal relation between a factor on an outcome would be interventional studies (Wang et al., 2011). However, this study did not conduct corresponding interventional studies on SCD patients. Second, known conditions that could affect the development of AD, such as family history of depression or dementia and an individual’s alcohol consumption could not be assessed. Third, from the perspective of statistical analysis, smaller number of participants may not be a good indicator of the true distribution of CFs. Fourth, the number of CFs is a relatively primitive statistical parameter, and interactions and activities between friends more likely seem to be key factors affecting brain function and individual behavior. Additionally, the number of friends is a very subjective data given by the subjects, and we could not analyze whether other people’s evaluation of the relationship with the subjects will affect the results, that is, there is a lack of objective evaluation of the subjects by other people. Fifth, although a single scale is significant, the diagnosis of SGD and SCD is more the result of multiple factors, such as family, marriage, self-conscious economic status, and physical health. Sixth, despite having a similar mean number of CFs, the correlation was not present in NC group, likely due to floor effects for depressive symptoms. Finally, our statistical analysis and established model were only conclusions drawn from existing data, and both SGD and SCD could be complex physiological and pathological processes with multiple factors. However, despite these limitations, our study provided a direction for the early prevention of AD from the perspective of brain function.

## Conclusion

In conclusion, this study explored the neural mediating role of the brain between CFs, SGD, and SCD in the elderly. We observed that the FFG.R may mediate social relationships and SGD through emotion recognition, and the abnormality of the SOG.R may be a key factor in the CD caused by depression. The results confirmed that a significant association existed between the number of CFs and SCD.

## Data availability statement

The raw data supporting the conclusions of this article will be made available by the authors, without undue reservation.

## Ethics statement

The studies involving human participants were reviewed and approved by the Xuanwu Hospital Review Committee and the Medical Research Ethics Committee. The patients/participants provided their written informed consent to participate in this study.

## Author contributions

ZZ and GL contributed to conception and design of the study. ZZ organized the database, performed the statistical analysis, wrote the first draft of the manuscript, and wrote sections of the manuscript. All authors contributed to manuscript revision, read, and approved the submitted version.
