# Corrigendum: Relationship among number of close friends, subclinical geriatric depression, and subjective cognitive decline based on regional homogeneity of functional magnetic resonance imaging data

**DOI:** 10.3389/fnagi.2022.1112384

**Published:** 2022-12-21

**Authors:** Zhao Zhang, Guangfei Li, Zeyu Song, Ying Han, Xiaoying Tang

**Affiliations:** ^1^Department of Biomedical Engineering, School of Life Sciences, Beijing Institute of Technology, Beijing, China; ^2^Department of Psychiatry, Yale University School of Medicine, New Haven, CT, United States; ^3^Department of Neurology, Xuanwu Hospital of Capital Medical University, Beijing, China

**Keywords:** number of close friends, subjective cognitive decline, regional homogeneity, mediation effect, subclinical geriatric depression

In the published article, there was an error in the **Funding** statement. The funding details were incorrectly written as “National Natural Science Foundation of China (U20A20388).” The corrected **Funding** statement appears below.

