# Supplementary material for: Relationship among number of close friends, subclinical geriatric depression, and subjective cognitive decline based on regional homogeneity of functional magnetic resonance imaging data
Source: Front Aging Neurosci. 2022 Sep 23;14:978611. doi: 10.3389/fnagi.2022.978611 (PMC9541299; doi:10.3389/fnagi.2022.978611)
Supplement: Supplementary file 1 [file Data_Sheet_1.docx]

Supplementary Material

# Data collection parameters

All participants were scanned on an integrated simultaneous 3.0 Tesla TOF PET/MR (SIGNA PET/MR, GE Healthcare, Milwaukee, WI, USA). The rs-fMRI scan lasted 8 minutes. 3D BRAVO T1-weighted sagittal images were obtained using the following parameters: SPGR sequence, repetition time (TR) = 6.9 ms, echo time (TE) = 2.98 ms, flip angle (FA) = 12°, inversion time (TI) = 450 ms, field of view (FOV) = 256×256 mm^2^, matrix = 256×256, gap=0, slice thickness = 1 mm, slice number = 192, and voxel size = 1×1×1 mm^3^. Single-shot gradient-echo EPI sequence, TR = 2000ms, TE =30 ms, FA = 90°, FOV = 224×224mm^2^, data matrix = 64×64, gap = 1.0 mm, slice thickness = 4.0 mm, slice number = 28, slice order = interleaved, and voxel size = 3.5×3.5×4 mm^3^.

# Clinical and neuropsychological assessments

All participants completed a conventional clinical evaluation that included a review of their health and family history, current medicines, physical examination, and standard blood tests, as well as evaluation using a battery of neuropsychological tests.

The neuropsychological battery included tests of general cognitive ability (Chinese version of Mini Mental State Examination (MMSE), memory and executive function screening instrument (MES), 15-item short form of the Geriatric Depression Scale (GDS) and 9-item subjective cognitive decline questionnaire (SCD-9). Cognitive testing was performed by trained neuropsychologists.

# KCC-ReHo Algorithm

For a given voxel, $R_{i}$(*i*=1,…,*n*) was denoted as the ranks of its R-fMRI BOLD time series and *n* as the number of time points. KCC-ReHo is defined as

$$\text{W}=\frac{\sum_{i=1}^{n} (R_{i})^{2}-n(\overline{R})^{2}}{\frac{1}{12}K^{2}(n^{3}-n)}=12\frac{\sum_{i=1}^{n} (\overline{R_{i}})^{2}}{(n^{3}-n)}-3\frac{(n+1)}{n-1}$$

where *K* is the number of neighbors (including the voxel, e.g., a total 27 voxels used in this study) of the voxel, $\overline{R}_{i}$is the mean rank across its neighbors at the *i*-th time point, and $\overline{R}$is the overall mean rank across all neighboring voxels and time points.

# Supplementary Figures


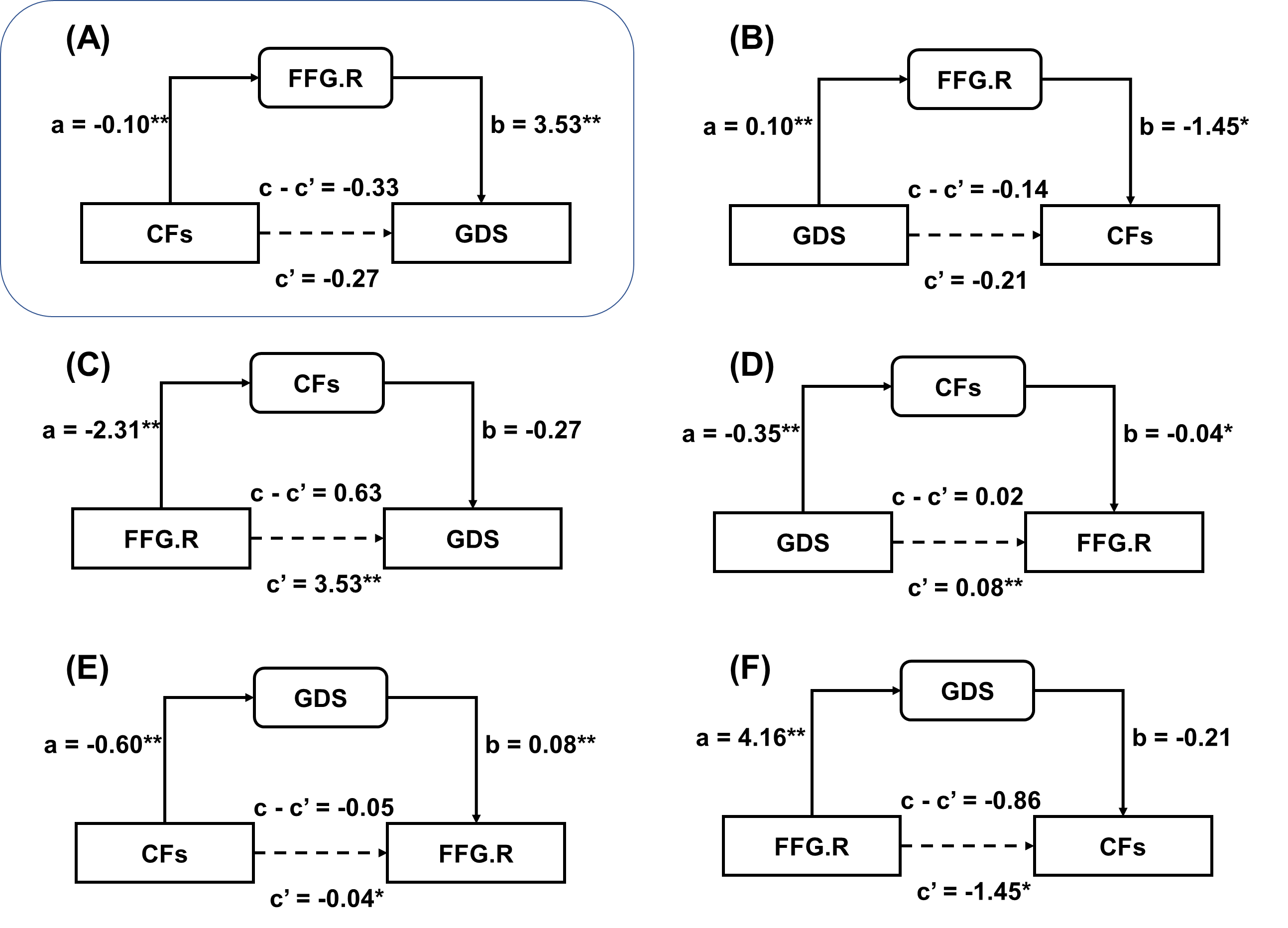


**Supplementary Figure S1.** Results of mediation analysis between CFs, FFG.R and GDS. A was the only substantial (and complete) mediated relationship. There were no substantial mediated relationships in B, C, D, E, and F models.


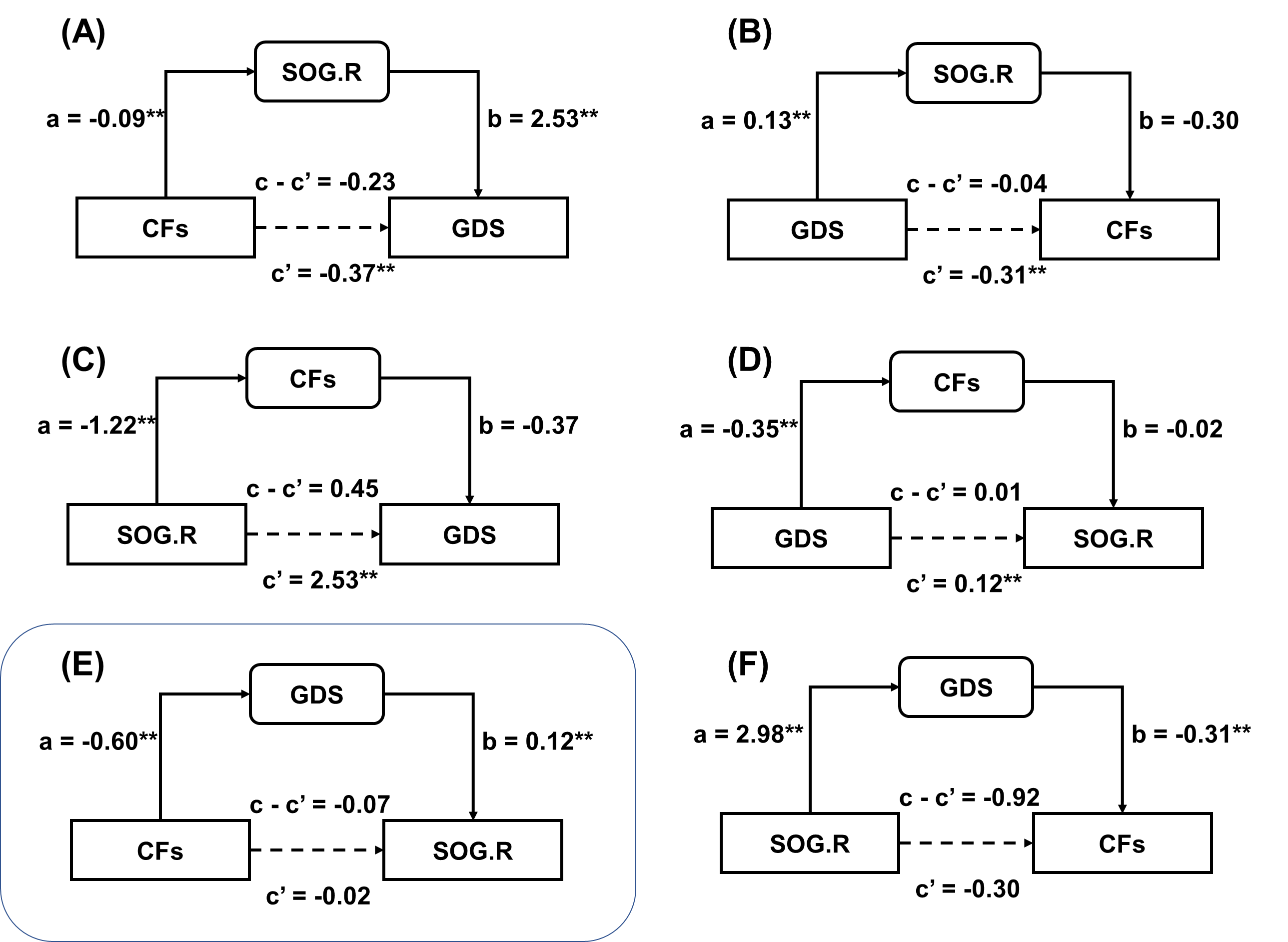


**Supplementary Figure S2.** Results of mediation analysis between CFs, SOG.R and GDS. E and F were substantial (and complete) mediated relationship; There were no substantial mediated relationships in A, B, C, and D models. E was selected because the causal relationship between CFs and GDS corresponds to Figure S1 (A)


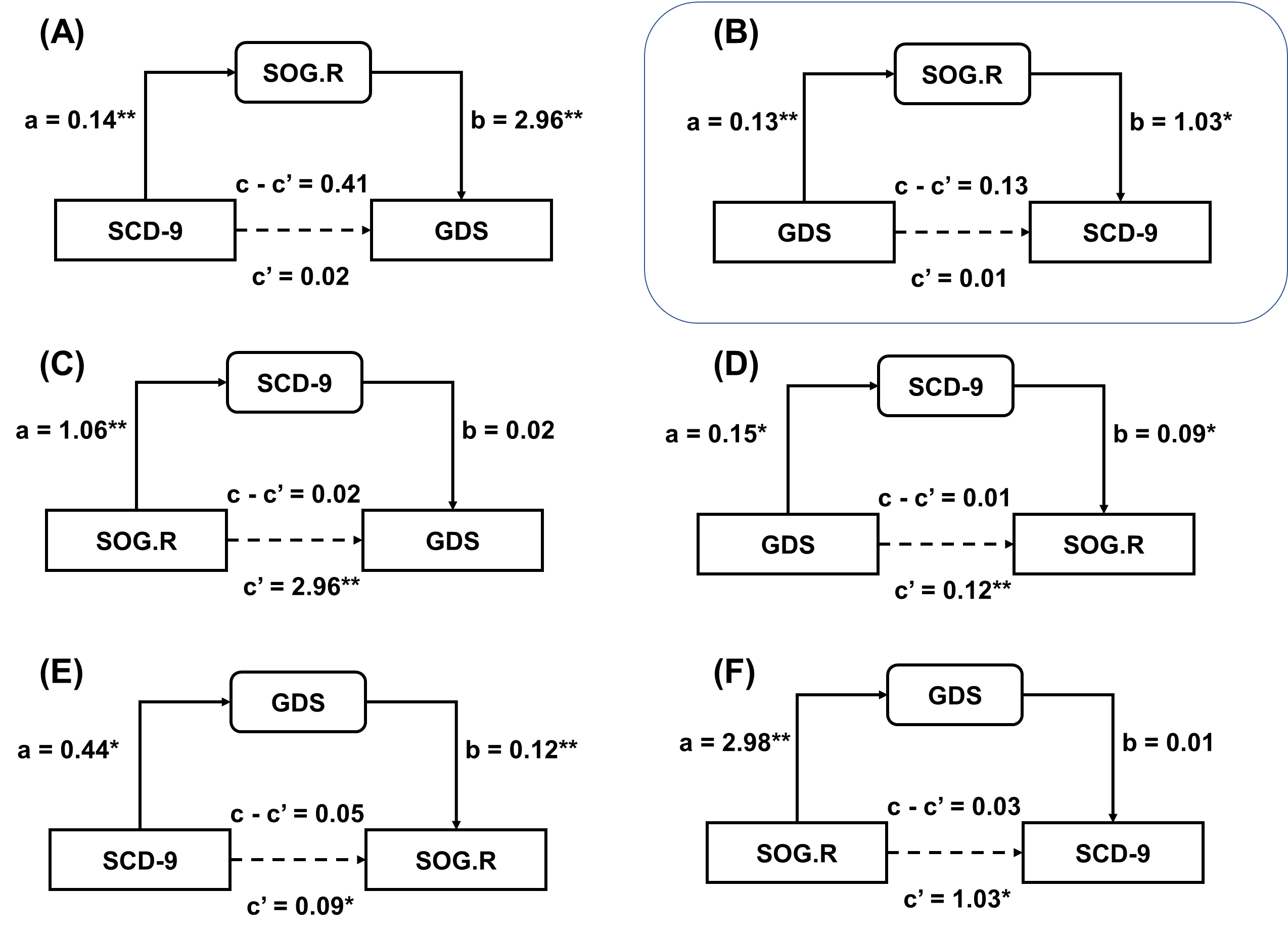


**Supplementary Figure S3.** Results of mediation analysis between GDS, SOG.R and SCD-9. A and B were substantial (and complete) mediated relationship; There were no substantial mediated relationships in C, D, E, and F models. B was selected because the causal relationship between GDS and SOG.R corresponds to Figure S2 (E)
